# The triangle of inequality in dental services: arguments for a new conceptual framework

**DOI:** 10.1186/s13690-022-00826-1

**Published:** 2022-02-18

**Authors:** Arash Ghanbarzadegan, Peivand Bastani, Madhan Balasubramanian, David Brennan, Lisa Jamieson

**Affiliations:** 1 Australian Research Centre for Population Oral Health (ARCPOH), Adelaide Dental School, The University of Adelaide, Adelaide, SA Australia; 2 Health Human Resources Research Centre, School of Health Management and Medical Informatics, Shiraz University of Medical Sciences, Shiraz, Iran; 3 School of Dentistry, Faculty of Health and Behavioural Sciences, University of Queensland, Herston, Queensland Australia; 4 Health Care Management, College of Business, Government and Law, Flinders University, Bedford Park, SA Australia; 5 Menzies Centre for Health Policy and Economics, School of Public Health, Faculty of Medicine and Health, The University of Sydney, Sydney, NSW Australia

**Keywords:** Access, Utilisation, Provision, Oral and dental health

## Abstract

This short communication paper aimed to compile the main determinants of inequality in dental services by distinguishing between access, utilisation, and provision of dental services. Recent findings integrated, and a dedicated conceptual framework entitled “Triangle of inequality in dental services” has been suggested. These can contribute a rich knowledge in this area and open a new window for policymakers and researchers to seek applied interventions to decrease inequality and improve access and utilisation in communities. This paper aims to synthesise the available evidence and add value to the scope. It highlights a dedicated concept for inequality in dental services beyond other areas of public health.

## Background

Oral health is integral to general health and wellbeing and is considered a global public health concern, particularly in many low- and middle-income countries (LMICs) [1]. Considering that oral disease is still a major public health burden worldwide with the greater global burden on the deprived and poor population [2], integrating oral health to general health globally can be among health policymakers’ interests [3]. Moreover, the direct and lasting impact of oral health on general health makes the policymakers prioritise access to dental services by the whole community at the top of agenda-setting.

Lack of integration between oral health and global public health with a population health approach can lead to many health concerns. Inequality in providing and utilising dental services can worsen these concerns. According to the evidence, many social, economic, cultural and environmental determinants at the macro and micro levels along with meso elements related to insurance, providers and policies and practices are among the determinants that lead to inequality in the area of oral health in LMICs [4]. Inequality in utilisation of dental services even in developed countries is severe and influenced by many factors. Individual, social, economic, and cultural determinants are the main determinants of inequality in utilisation of dental services. In addition to this, health policies and availability of services are indicators of provision of dental services that lead to inequalities in such services [5]. For instance, changes in oral health policies during the COVID-19 pandemic with greater emphasis on providing emergency services at the peak of the outbreak, along with restricting non-emergency dental services, have significant impacts on the population’s access and utilisation of dental services [6].

Evidence emphasises the necessity of policymakers’ particular attention to appropriate access to dental services to decrease inequalities and improve the oral health of a given population. Despite identifying the above determinants, the nature of dental services is uniquely different from public health services. In other words, although oral health should be considered as part of global public health, because of the different nature of oral health and in particular dental services, inequality in dental services can be intensified and should be regarded as a serious concern. Therefore, it is essential that policymakers focus on a dedicated model to better understand inequality mechanisms in dental services. Some reasons can justify the necessity of such a model.

In the dental literature, access, utilisation and provision of dental services are terms often used interchangeably. However, to better address the determinants of inequality in dental services, it is essential to distinguish between these concepts. In health sciences, service utilisation is defined as the individual's preventive or curative service use; service provision deals with the process of providing services according to the available resources (human resources, physical capital and consumables) [5, 7]. Access to health services is the timely use of these services to achieve the best health outcomes both at individual and population levels [8].

Dental services are uniquely different from other public health and primary health care services. Dental services often have higher costs with limited comprehensive insurance coverage, so the out-of-pocket payments are relatively high for dental services. This creates a model of care that favours emergency dental visits. A cycle of delayed referrals, specialised needs and higher cost of these specialised services increases the gap in utilisation and therefore leads to a worsening oral health status at the population level [5].

The impact of community diversities in cultural, economic, social and health literacy on the utilisation of dental services should not be under-estimated [9]. Although this is a global issue, it should be formulated and customised according to different contexts, policymakers motivations, priorities and severity of the issue for any context.

A conceptual model is helpful in highlighting the principal elements and strategic points that better facilitate timely and appropriate dental service provision. Such a model should integrate different concepts of access, utilisation and provision of dental services. It clarifies that more appropriate provision of quality services affects higher access and broader utilisation of the services. In addition, different aspects of access, such as physical, geographical, cultural and financial aspects, along with the acceptability of the services [10], lead to higher dental service utilisation, and as a result, a reduction in inequalities in dental services.

### Conceptual framework

**Fig. 1:**
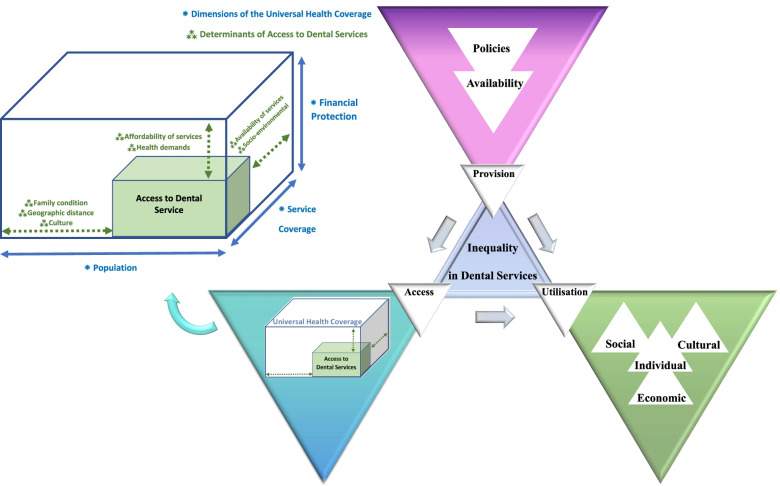
Triangle of Inequality in dental services, introduced by Ghanbarzadegan et al. [5, 10]

Ghanbarzadegan et al. (2021) previously introduced the *“Triangle of Inequality”* in dental services concept (Fig. 1) [5, 10]. As illustrated in Fig. 1, provision of dental services in the top of this hierarchy influences access and utilisation of services. In this framework, determinants of access to dental services are aligned with the Universal Health Coverage (UHC) dimensions, with the key to increasing access to dental services being to reduce the existing gap in dental services universal coverage. Each small triangle is also directed to another triangle to show how these determinants are intertwined. For example, in the framework of service provision, oral health policies influence the availability of services and these would define access to dental services from different perspectives such as availability, affordability or even geographic access. Providing need-based dental services at the affordable and acceptable prices improves the access to these services. Simultaneously, the provision of oral and dental services is affected by the availability of the services and oral and dental health policies. According to the triangle, if the services are provided and are accessible to the population, inequality will decrease only by developing service utilisation. This third item will be fulfilled considering the individual, cultural, social and economic determinants of the population.

Policymakers may move to a trade-off among these determinants according to the priorities, community’s conditions, financial capability (e.g., GDP per capita allocated specifically for dental health provision), basic health benefit packages and insurance coverage of dental services. Also, in the access aspect of the model, the supply chain, which indicates the dental service providers, availability of human resources and equipment, can be effective along with the demand side of the population and its demographic characteristics as well other determinants of utilisation, provision and access to services previously discussed.

## Conclusion

In conclusion, due to the unique nature of dental services and their differences with other health services, the lack of clear definitions of various dimensions of inequality in services and inappropriate and mistakenly interchangeable use of access, utilisation and provision concepts, there is a need to redefine these dimensions in dental services. The “triangle of inequality” as a conceptual model in dental services may assist in ameliorating inequalities in dental services by clarifying the concepts mentioned above and their determinants.

## Data Availability

Are available from the corresponding author on reasonable request.

## Abbreviation

LMIC: Low- and middle-income countries

## References

[1] Peres MA, Macpherson LM, Weyant RJ, Daly B, Venturelli R, Mathur MR (2019). Oral diseases: a global public health challenge. Lancet.

[2] Petersen PE, Bourgeois D, Ogawa H, Estupinan-Day S, Ndiaye C (2005). The global burden of oral diseases and risks to oral health. Bull World Health Organ.

[3] Jin L, Lamster I, Greenspan J, Pitts N, Scully C, Warnakulasuriya S (2016). Global burden of oral diseases: emerging concepts, management and interplay with systemic health. Oral Dis.

[4] Bastani P, Mohammadpour M, Mehraliain G, Delavari S, Edirippulige S (2021). What makes inequality in the area of dental and oral health in developing countries? A scoping review. Cost Effect Resour Allocation.

[5] Ghanbarzadegan A, Bastani P, Luzzi L, Brennan D (2021). Inequalities in utilization and provision of dental services: a scoping review. Syst Rev.

[6] Jiang CM, Duangthip D, Auychai P, Chiba M, Folayan MO, Hamama HHH (2021). Changes in oral health policies and guidelines during the COVID-19 pandemic.

[7] Carrasquillo O, Gellman MD, Turner JR (2013). Health care utilization. Encyclopedia of behavioral medicine.

[8] Gulliford M, Figueroa-Munoz J, Morgan M, Hughes D, Gibson B, Beech R (2002). What does' access to health care'mean?. J Health Serv Res Policy.

[9] Evans DB, Hsu J, Boerma T. Universal health coverage and universal access. Bulletin of the World Health Organization. 2013;91:546–A. 10.2471/BLT.13.125450 PMC3738317 23940398

[10] Ghanbarzadegan A, Balasubramanian M, Luzzi L, Brennan D, Bastani P (2021). Inequality in dental services: a scoping review on the role of access toward achieving universal health coverage in oral health. BMC Oral Health.

